# Sensorimotor Memory Biases Weight Perception During Object Lifting

**DOI:** 10.3389/fnhum.2015.00700

**Published:** 2015-12-23

**Authors:** Vonne van Polanen, Marco Davare

**Affiliations:** ^1^Motor Control Laboratory, Movement Control and Neuroplasticity Research Group, Biomedical Sciences Group, Department of KinesiologyKatholieke Universiteit Leuven, Leuven, Belgium; ^2^Sobell Department of Motor Neuroscience and Movement Disorders, UCL Institute of NeurologyLondon, UK

**Keywords:** grasping, lifting, sensorimotor memory, weight perception, motor control

## Abstract

When lifting an object, the brain uses visual cues and an internal object representation to predict its weight and scale fingertip forces accordingly. Once available, tactile information is rapidly integrated to update the weight prediction and refine the internal object representation. If visual cues cannot be used to predict weight, force planning relies on implicit knowledge acquired from recent lifting experience, termed sensorimotor memory. Here, we investigated whether perception of weight is similarly biased according to previous lifting experience and how this is related to force scaling. Participants grasped and lifted series of light or heavy objects in a semi-randomized order and estimated their weights. As expected, we found that forces were scaled based on previous lifts (sensorimotor memory) and these effects increased depending on the length of recent lifting experience. Importantly, perceptual weight estimates were also influenced by the preceding lift, resulting in lower estimations after a heavy lift compared to a light one. In addition, weight estimations were negatively correlated with the magnitude of planned force parameters. This perceptual bias was only found if the current lift was light, but not heavy since the magnitude of sensorimotor memory effects had, according to Weber’s law, relatively less impact on heavy compared to light objects. A control experiment tested the importance of active lifting in mediating these perceptual changes and showed that when weights are passively applied on the hand, no effect of previous sensory experience is found on perception. These results highlight how fast learning of novel object lifting dynamics can shape weight perception and demonstrate a tight link between action planning and perception control. If predictive force scaling and actual object weight do not match, the online motor corrections, rapidly implemented to downscale forces, will also downscale weight estimation in a proportional manner.

## Introduction

Perceiving and handling objects are inherently linked. To grasp, move or use an object accurately, its sensed physical properties must rapidly be integrated into the motor plan. For instance, while the grasp aperture is proportional to the size of the object (Jeannerod et al., 1995; Castiello, 2005), fingertip forces that are used to lift it are scaled according to the expected weight and frictional properties of the object in order to ensure a stable grasp and avoid slips (Johansson and Westling, 1984). Fingertip force planning based on an expectation of the object weight is crucial, as feedback mechanisms are generally too slow and will result in a less smooth lift (Johansson and Westling, 1984, 1988). Previous lift experience with the object is used to build an internal model that can be used to predict the object weight and thus scale fingertip forces accordingly. In the absence of cues allowing weight prediction, it has been shown that force scaling is influenced by the object weight or frictional properties in the preceding lifts (Johansson and Westling, 1984, 1988). This effect of lift history on force scaling has been termed sensorimotor memory, can be found on a trial-by-trial basis (Johansson and Westling, 1988; Chouinard et al., 2005) and is also reflected in the corticospinal excitability (Loh et al., 2010). Precise force scaling is classically assessed by quantifying the force rate increase just after object contact and before lift-off (Loh et al., 2010; Baugh et al., 2012). For example, if a heavy object is lifted in the previous trial, force rates in the current lift will be larger compared to when a light object is previously lifted.

In return, acting upon an object provides additional sensory inputs that enhance perceptual information about its physical properties. Here, we refer to “perception” as explicit knowledge about an object property. Knowledge about the material, weight or inertia of an object can be acquired by touching and lifting it. Perception of weight has been studied extensively in psychophysical studies (Jones, 1986). Discrimination abilities follow Weber’s law, that is, just noticeable differences depend on the intensity of the stimulus. Weight perception is, however, not always veridical, as shown by several weight illusions (Buckingham, 2014) of which the size-weight illusion (Charpentier, 1891) is the most notable and investigated one.

When objects vary in size, a weight expectation based on the size can be made if the object density is constant. In the size-weight illusion, smaller objects are perceived to be heavier than larger ones even though they actually weigh the same (Charpentier, 1891). In the first trials, fingertip forces are scaled to the “expected” object weight, based on the size (i.e., larger force scaling for the large object). The mere expectation based on object size is enough to create the illusion (Buckingham and Goodale, 2010). However, after a few trials, forces are accurately scaled to the actual object weight (i.e., equal force scaling for both objects), whereas the perceptual illusion remains (Flanagan and Beltzner, 2000; Grandy and Westwood, 2006). Flanagan and Beltzner (2000) argued that the grasping parameters are determined by sensorimotor memory. This separate adjustment of fingertip force scaling and illusionary perception suggests a dissociation between the control of action and perception. In the visual system, the dual-stream theory assumes a different neural processing of visual sensory input for action (“where/how”) and perceptual related tasks (“what”) in the dorsal and ventral stream, respectively (Goodale and Milner, 1992). Such a separation between brain areas for the processing of spatial and identity information has also been found in other modalities (Romanski et al., 1999; Reed et al., 2005; Dijkerman and de Haan, 2007).

Thus, if the control of action and perception is strictly separated, perceptual estimates should not be influenced by how an object is lifted. Previous research indicates that this is not true. For instance, if less grip force (GF) is needed to grasp an object due to a higher friction (Flanagan et al., 1995), or because more fingers or a higher contact area can be used (Flanagan and Bandomir, 2000) the object is perceived as lighter. Moreover, if a higher grip force (resulting in a larger safety margin) is consciously used to lift an object, the rating performance to differentiate between weights decreases compared to when a normal grip is used (Ellis and Lederman, 1999). It is noteworthy that these studies investigated whether an altered grip force throughout the lifting movement, i.e., during both the dynamic and static phases, can affect weight perception. Hence, it is still unclear whether changes in force scaling that only occur within the initial dynamic phase would bias perception. Here, we took advantage of sensorimotor memory effects in order to manipulate experimentally the force scaling. Sensorimotor memory gives rise to an implicit “expectation” about the upcoming weight and only influences the dynamic phase of the lifting motion. Since we know this specific phase reflects motor planning based on the expected weight, any effects of force scaling on weight perception would demonstrate a tight link between the planning of actions and perception, two systems that were long thought to be independent.

To address the influence of sensorimotor memory on weight perception, we compared lifts preceded by light or heavy objects and quantified force scaling and object weight rating for each trial (Experiment 1a). We hypothesized participants would assign different weight estimates for lifts of a given object that was preceded by a light compared to a heavy object. In a follow-up experiment (Experiment 1b), we experimentally increased the magnitude of sensorimotor memory effects by lengthening the same weight trial history. Here, we hypothesized that larger effects on force scaling would in turn lead to larger perceptual weight biases. In order to examine the effect of trial history on weight estimates in the absence of sensorimotor memory, we performed a passive weight perception task (Experiment 2) where forces were applied on the participants’ resting hand. In this task, we expected no perceptual biases.

## Materials and Methods

### Participants

A total of 28 healthy participants took part in the study. Ten participants took part in Experiment 1a with a mean age of 30.4 years (age range of 21–41 years, 6 females, all right-handed). Another 10 different subjects participated in Experiment 1b, with a mean age of 22.4 years (age range 19–27 years, 4 females, all right-handed). Finally, in Experiment 2, eight other participants took part with a mean age of 29.9 years (range 23–34 years, 4 females, 6 right-handed). Before the start of the experiments, they all provided informed consent. Experiments were performed in accordance with principles as stated in the declaration of Helsinki and were approved by the local ethical committee of the Faculty of Biomedical Sciences, Katholieke Universiteit Leuven.

### Apparatus

**Figure 1:**
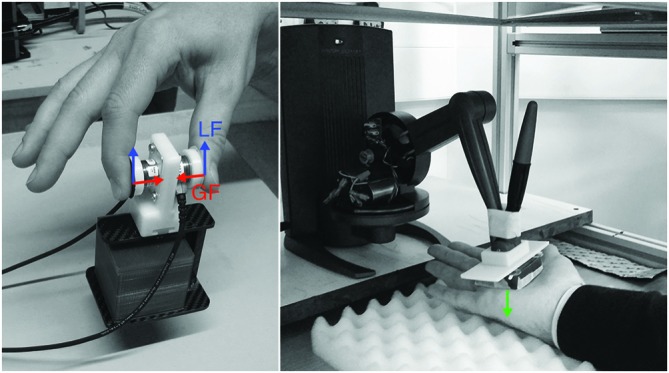
**Experimental set-up in Experiment 1 (left) and Experiment 2 (right).** In Experiment 1, subjects had to grasp and lift a manipulandum with force sensors measuring the grip (GF, red) and load forces (LF, blue). A small carbon fiber basket was attached underneath the manipulandum to allow placement of different weights (100, 300 or 500 g). In Experiment 2, a force feedback robot (Geomagic Touch X) was used to apply forces (1, 3 or 5 N; green arrow) passively on the subjects’ right hand.

A grip-lift manipulandum consisting of two 3D force-torque sensors (Nano17, ATI Industrial Automation, Apex, NC, USA) was attached to a custom-made carbon fiber basket in which different objects (cubes) could be placed (Figure 1, left). The weight of the basket underneath the manipulandum was perfectly balanced, using a slider. The graspable surface (17 mm diameter and 45 mm apart) of the force sensors was covered with fine sandpaper (P600) to increase friction. The forces in three directions were sampled at 1000 Hz. The objects were 3D-printed cubes of 5 × 5 × 5 cm, filled with different amounts of lead particles to create weights of 100, 300 and 500 g. Note that the loads the participants lifted also included the combined weight of the manipulandum and basket, which had a total weight of 120 g. To prevent visual cues, the cubes were hidden under a paper cover. The manipulandum was placed behind a transparent switchable screen (Magic Glass), which was either opaque or transparent.

The experimental set-up used in Experiment 2 is pictured in Figure 1 (right). A Geomagic Touch X Haptic Device (3D systems, Rock Hill, SC, USA) was used to exert a normal force (~1, 3 or 5 N) onto the palm of the participants’ right hand. The end of the device arm was fixed to a plastic plate with a size of about 5 × 5 cm. The forces were applied instantly on the hand.

### Experiment 1: Grip-Lift Task and Procedure

In the first experiment, participants were instructed to grasp and lift the manipulandum with the thumb and index finger placed on each force sensor. They had to lift it at a comfortable pace up to a height of 2 cm, hold it steady for a few seconds and then release it back on the table. The trial started when the switchable screen turned transparent, accompanied by a beep indicating participants could initiate the grasp. The screen remained transparent for 3 s and then turned opaque again indicating the object had to be replaced on the table for the next trial. Weight perception judgments were acquired using the method of magnitude estimation (Zwislocki and Goodman, 1980): just after object release, participants were asked to assign a number best representing the perceived weight, based on an arbitrary numerical scale (with no explicit upper or lower limit).

The objects were presented in a semi-randomized order. In Experiment 1a, sensorimotor memory was probed using the four possible two-trial sequences, namely a current *light* lift preceded by a light object (L*L*) or heavy (H*L*), or a current *heavy* lift preceded a light object (L*H*) or heavy (H*H*). These four conditions were presented 10 times each. In Experiment 1b, we sought to investigate the effect of a longer trial history. Block length was increased so as to include 10 times the following three-trial sequences: light-light-*light* (LL*L*), heavy-heavy-*light* (HH*L*), light-light-*heavy* (LL*H*) and heavy-heavy-*heavy* (HH*H*). The light object was 100 g, the heavy object 500 g. An intermediate object of 300 g was presented five times in Experiment 1a and 10 times in Experiment 1b as a dummy trial to make object weight presentation less repetitive (about 10% of the trials). The total lifted weight also included the mass of the manipulandum (120 g). The total number of trials was 51 and 100 in Experiments 1a and 1b, respectively. This number consists of 40 analyzed trials (four conditions repeated 10 times) and unanalyzed trials (dummy trials, trials directly after dummy trials and trials that only served as preceding lifts in longer sequences). To minimize the total trial number, a single trial could sometimes serve as a preceding lift as well as an analyzed lift. A trial lasted 3 s and participants had full view of the object during this time (i.e., the screen was transparent). Before the experiment, participants performed practice trials with an object of 200 g.

### Experiment 2: Passive Estimation Task and Procedure

In Experiment 2, a control experiment was performed where participants performed weight estimations, but without actively lifting the object. The goal of this experiment was to investigate the presence of a perceptual history effect in the absence of active force control. Participants were instructed to rest their right hand flat on the table, with the palm up. A haptic device exerted a normal force onto their hand palm for 3 s. They were told not to move their hand during the trial. Participants were asked to estimate the magnitude of the object weight on an arbitrary numerical scale, as in Experiment 1. The weight presentation sequence was the same as in Experiment 1a, where the four possible sequences of 2 weights (L*L*, H*L*, L*H* and H*H*) were presented 10 times each in a semi-randomized order. Forces of 1 N were used for light objects, 5 N for heavy objects and 3 N for dummy trials (10%). The start of force application was indicated by an auditory beep.

### Force and Perceptual Parameters

In Experiments 1 and 2, participants’ weight ratings were normalized by dividing each trial answer by the average of all perceptual estimates for each participant. Force parameters and perceptual ratings were averaged over trials in the four conditions: LL, HL, LH and HH (Experiments 1a, 2) and LLL, HHL, LLH and HHH (Experiment 1b). Dummy trials or trials that followed dummy trials were not analyzed. Three (0.75%), two (0.5%) and five (1.6%) trials were removed from analysis due to technical errors in Experiments 1a, 1b and 2, respectively.

**Figure 2:**
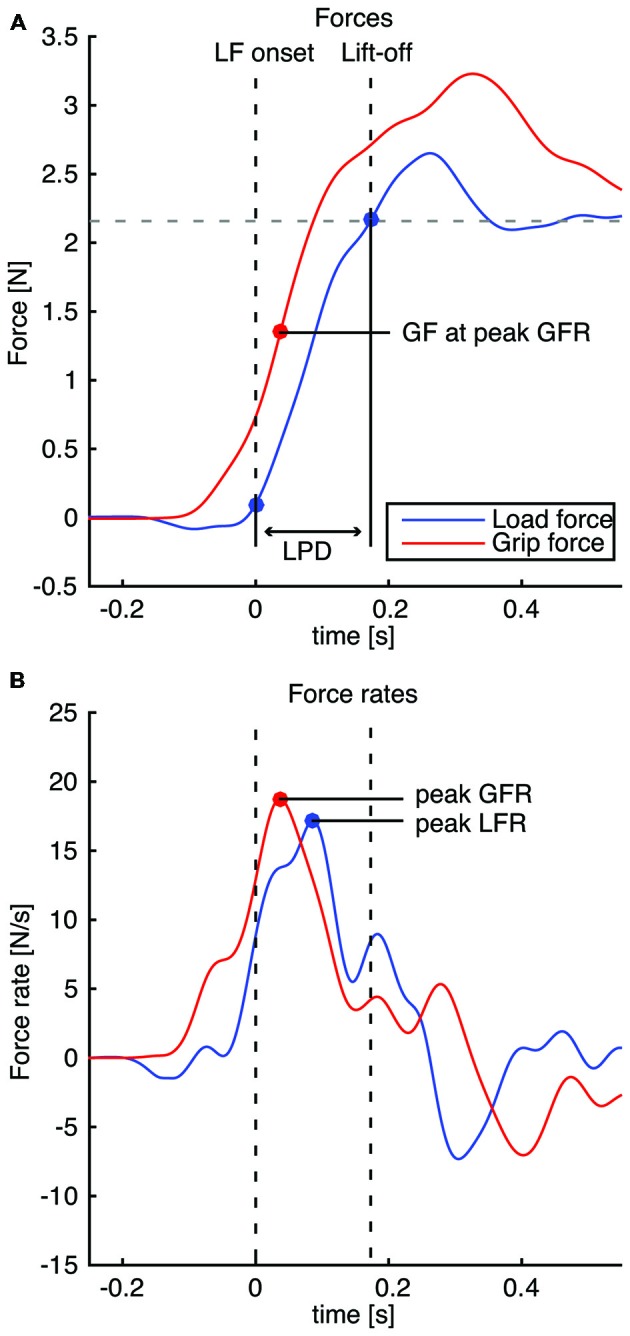
**Illustration of the measured fingertip force parameters: load phase duration (LPD), peak grip force rate (peak GFR), peak load force rate (peak LFR) and GF at peak GFR.** Vertical dashed lines indicate LF onset and lift-off. **(A)** Red and blue solid lines indicate the recorded GF and LF respectively; the horizontal dashed line represents the static load force for the light object (including the basket). **(B)** Force rates. Note all traces are aligned to LF onset.

In Experiment 1, baseline force sensor levels were measured before the experiment started when the manipulandum was placed stationary on the table. These baseline values were subtracted from the data to remove the offset and voltages were converted to Newtons. Force signals were filtered using a bidirectional 2nd-order Butterworth filter with a cut-off frequency of 10 Hz. The grip force (GF) was the average of the horizontal forces perpendicular to the graspable surface of both force sensors. The load force (LF) was defined as the sum of the vertical forces tangential to the graspable surface of both force sensors (Figure 1). The grip force rate (GFR) and the load force rate (LFR) were the differentiated GF and LF, respectively. GF and LF onsets were determined when force signals reached a threshold of 0.1 N after which a minimum of 0.8 N had to be reached to control for small non-meaningful force fluctuations. The variables of interest were the peak force rates (peak GFR and peak LFR), the GF value at peak GFR and the duration of the loading phase (LPD) and are illustrated in Figure 2. Because we were interested in the early stages of force planning, peak force rates were defined as the first peak that was higher than 70% of the maximal force rate. The LPD was defined as the time delay between LF onset and the first time LF overcame the static load. Static load force values were measured in a separate session for each weight by the first author (2.2, 4.2 and 6.2 N, for the light, intermediate and heavy object, respectively), which included the weight of the cube, manipulandum and basket. The GF value at peak GFR was calculated to quantify the sensorimotor memory effect on the actual force before the influence of feedback mechanisms. This value was used to compare the magnitude of the sensorimotor memory effect to the lifted weights.

### Statistical Analyses

We analyzed the effect of sensorimotor memory on force and perceptual parameters by comparing trials preceded by either heavy or light objects. These analyses were performed separately for light or heavy lifts (Experiments 1a, b) or perceptual trials (Experiment 2). In other words, sensorimotor memory effects on a current *light* (*L*) lift were tested by comparing H*L* vs. L*L* (or HH*L* vs. LL*L* in Experiment 1b) conditions whereas sensorimotor memory effects on a current *heavy* (*H*) lift were tested by comparing L*H* vs. H*H* (or LL*H* vs. HH*H* in Experiment 1b) conditions. Comparisons were assessed using paired *t*-tests with an *α*-value of 0.05.

To evaluate the trial-by-trial relationship between force parameters and weight perception, peak force rates and perceptual estimates were correlated within each subject. In conjunction with these within-subject correlation analyses, covariance analyses were performed on the weight ratings, with peak GFR or peak LFR as covariates and participants as fixed factors. Again, these covariance analyses were performed separately for *light* (H*L* and L*L* in Experiment 1a or HH*L* and LL*L* in Experiment 1b) and *heavy* lifts (L*H* and H*H* in Experiment 1a or LL*H* and HH*H* in Experiment 1b).

In a second between-subject analysis, we tested whether sensorimotor memory effects correlated with weight perception (Figure 4). Considering the low number of participants in Experiments 1a and 1b, data of these two experiments were pooled together. To do this, data of Experiment 1b were reanalyzed by calculating all variables grouped into the shorter two-trial sequences (e.g., L*L* instead of LL*L*). Then, we computed a force ratio (*X*-axis, Figure 4) by dividing GFR or LFR peak values of trials preceded by light lifts by trials preceded by heavy ones (i.e., L*L*/H*L* and L*H*/H*H*); a force ratio below 1 denoting an increasingly larger sensorimotor memory effect. Similarly, a perceptual ratio (*Y*-axis) was computed for weight ratings using the same formula as for force ratios (L*L*/H*L* and L*H*/H*H*). Here a ratio above 1 denotes a larger perceptual bias in weight estimation.

Finally, parameters measured in Experiment 1b were analyzed by grouping lifts in both two-trial and three-trial sequences in order to determine the effect of lengthening the trial sequence on the magnitude of sensorimotor memory and perceptual bias. For each variable (peak GFR, peak LFR, LPD, GF at peak GFR and weight ratings) and for light and heavy lifts separately, we quantified sensorimotor memory by computing ratios using the same formula as above, i.e., L*L*/H*L* and L*H*/H*H* for the two-trial sequences and compared them with ratios for the three-trial sequences (i.e., LL*L*/HH*L* and LL*H*/HH*H*, respectively). These ratios were compared with paired samples *t*-tests for light and heavy objects separately.

## Results

### Experiment 1a: Sensorimotor Memory Biases Weight Perception

**Figure 3:**
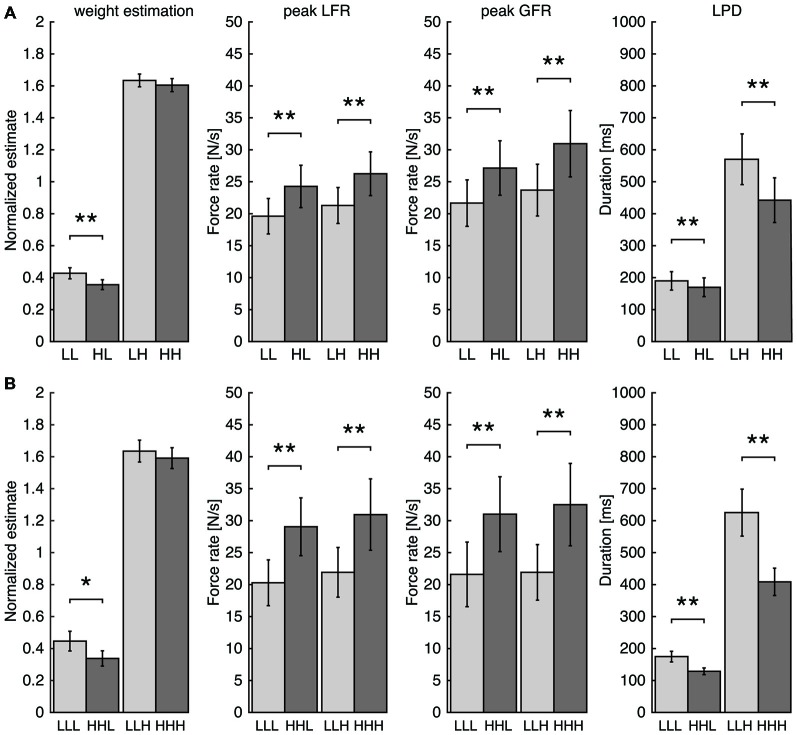
**Results for Experiment 1A (A) and Experiment 1B (B) for the normalized perceptual estimates, peak load force rates (peak LFR), peak grip force rates (peak GFR) and the load phase duration (LPD).** Bars represent the average of trial groups based on the weight sequence (light: L, heavy: H). Error bars represent standard errors of the mean. Note the effect of the previous lift on force parameters for both light and heavy objects whereas weight estimation was only affected for light objects. *p < 0.05, **p < 0.01.

The goal of Experiment 1 was to test whether previous lift history (i.e., sensorimotor memory) influenced weight estimation of the currently lifted object. In Experiment 1a (Figure 3A), only the directly preceding lift was taken into consideration and sequences of two trials were compared. A systematic sensorimotor memory effect was found for both light and heavy lifts. When a light lift was preceded by a heavy object (condition H*L*), higher peak GFR (*t*_(9)_ = −5.95, *p* < 0.001), higher peak LFR (*t*_(9)_ = −5.94, *p* < 0.001) and shorter LPD (*t*_(9)_ = 3.48, *p* = 0.007) were observed compared to when it was preceded by a light object (L*L*). Similarly, when a heavy lift was preceded by a light object (condition L*H*), peak GFR was lower (*t*_(9)_ = −4.41, *p* = 0.002), peak LFR was lower (*t*_(9)_ = −6.22, *p* < 0.001) and the LPD was longer (*t*_(9)_ = 8.07, *p* < 0.001) than when it was preceded by a heavy object (H*H*). GF values at peak GFR followed the same pattern (L*L*: 1.80 ± 0.26, H*L*: 2.33 ± 0.26, L*H*: 2.17 ± 0.22, H*H*: 2.63 ± 0.31; mean ± SEM) with significant differences for lifts of light (*t*_(9)_ = −6.83, *p* < 0.001) and heavy objects (*t*_(9)_ = −3.53, *p* = 0.006). These results are in line with previous findings showing that sensorimotor memory can bias the predictive scaling of force parameters when lifting a series of objects. Here, we took advantage of this sensorimotor memory effect to test whether a change in force scaling during the loading phase will in turn influence perceptual estimates about the object weight.

Interestingly, we also found an effect of trial history on object weight perception. Perceptual estimates were significantly different for the light object (*t*_(9)_ = 4.73, *p* = 0.001), but failed to reach significance for the heavy object (*t*_(9)_ = 0.86, *p* = 0.411; Figure 3A left). This indicates that the perception of light objects is influenced by the previous weight: the object feels lighter when a heavy object was previously lifted (H*L*) compared to when it was preceded by a light one (L*L*).

To estimate whether the peak force rates were correlated with the perceptual weight estimates on a trial-by-trial basis, a covariance analysis was performed. Here, a significant effect on the perceptual estimates was found for both peak GFR (*F*_(1,188)_ = 6.2, *p* = 0.014) and peak LFR (*F*_(1,188)_ = 14.0, *p* < 0.001) for the light lifts. The relationship between force and perceptual parameters was negative: lower weight estimations were associated with higher peak force rates. For the heavy lifts, no effect was found (peak GFR: *F*_(1,187)_ = 0.12, *p* = 0.73; peak LFR *F*_(1,187)_ = 0.09, *p* = 0.77). Individual relationships between force and perceptual parameters revealed mostly negative correlations with light lifts (8 out of 10 participants for peak GFR and 10 out of 10 for peak LFR). For heavy objects, correlation directions were more mixed (6 out of 10 participants negative for peak GFR and 7 out of 10 for peak LFR).

It is noteworthy that the absence of any perceptual bias for heavy lifts might be explained by the fact that the magnitude of the sensorimotor memory effect (GF at peak GFR rate difference: 0.53 and 0.46 N for light and heavy lifts, respectively) is drastically much smaller for heavy lifts (about 7%) vs. light lifts (about 24%) hence much less salient for inducing an effect on perception (see “Discussion” Section).

### Experiment 1b: Larger Sensorimotor Memory Effects Increase Weight Perception Bias

The goal of Experiment 1b was to experimentally manipulate the magnitude of the sensorimotor memory effect and test its impact on the weight rating bias. We expected a larger sensorimotor memory effect with a longer sequence of same weight lifts in the preceding trials. Such trials were too few to be analyzed in the data of Experiment 1a. However, a preliminary analysis showed that differences between lifts preceded by two light and two heavy trials seemed to increase for the force parameters as well as for perceptual estimates. Motivated by this observation, we purposely designed a new experiment (Experiment 1b) with longer, three-trial sequences of light and heavy objects (e.g., LL*L*, HH*L* etc.). As can be seen in Figure 3B, the results of this experiment were similar to Experiment 1a. For the force parameters, a sensorimotor memory effect was observed for light as well as heavy lifts. Lifts preceded by two heavy objects (HH*L* or HH*H*) showed a higher peak GFR (light: *t*_(9)_ = −7.85, *p* < 0.001; heavy: *t*_(9)_ = −4.82, *p* < 0.001), a higher peak LFR (light: *t*_(9)_ = −8.16, *p* < 0.001; heavy: *t*_(9)_ = −5.01, *p* < 0.001) and a shorter LPD (light: *t*_(9)_ = 6.38, *p* < 0.001; heavy: *t*_(9)_ = 6.31, *p* < 0.001) compared to lifts preceded by two light objects. The GF at peak GFR showed similar effects (LL*L*: 1.61 ± 0.24, HH*L*: 2.33 ± 0.28, LL*H*: 1.92 ± 0.29, HH*H*: 2.71 ± 0.38; mean ± SEM) with significant differences for light (*t*_(9)_ = −6.25, *p* < 0.001) and heavy lifts (*t*_(9)_ = −4.44*, p* = 0.002). In addition, we replicated our effect of trial history on weight perception. Perceptual estimates were lower if a light lift was preceded by two heavy objects (HH*L*) compared to two light objects (LL*L*; *t*_(9)_ = 2.96, *p* = 0.016). No significant perceptual effect was seen for the heavy object (*t*_(9)_ = 1.10, *p* = 0.30).

Within-subject analyses were performed to investigate the trial-by-trial correlations between force and perceptual parameters. A covariance analysis revealed that the peak GFR was related to the perceptual estimate within participants (light: *F*_(1,188)_ = 6.16, *p* = 0.014; heavy: *F*_(1,188)_ = 7.12, *p* = 0.008). The same result was found for the relationship between the peak LFR and the weight ratings (light: *F*_(1,188)_ = 18.6, *p* < 0.001; heavy: *F*_(1,188)_ = 7.49, *p* = 0.007). Again, this relation was negative where lower perceptual estimates were seen for higher force rates. For the individual participants, 9 out of 10 had negative correlations between weight ratings and peak GFR and 8 out of 10 with peak LFR for light lifts. For heavy lifts, negative correlations of weight ratings with peak force rates were observed in only 6 out of 10 participants for both peak GFR and peak LFR.

**Figure 4:**
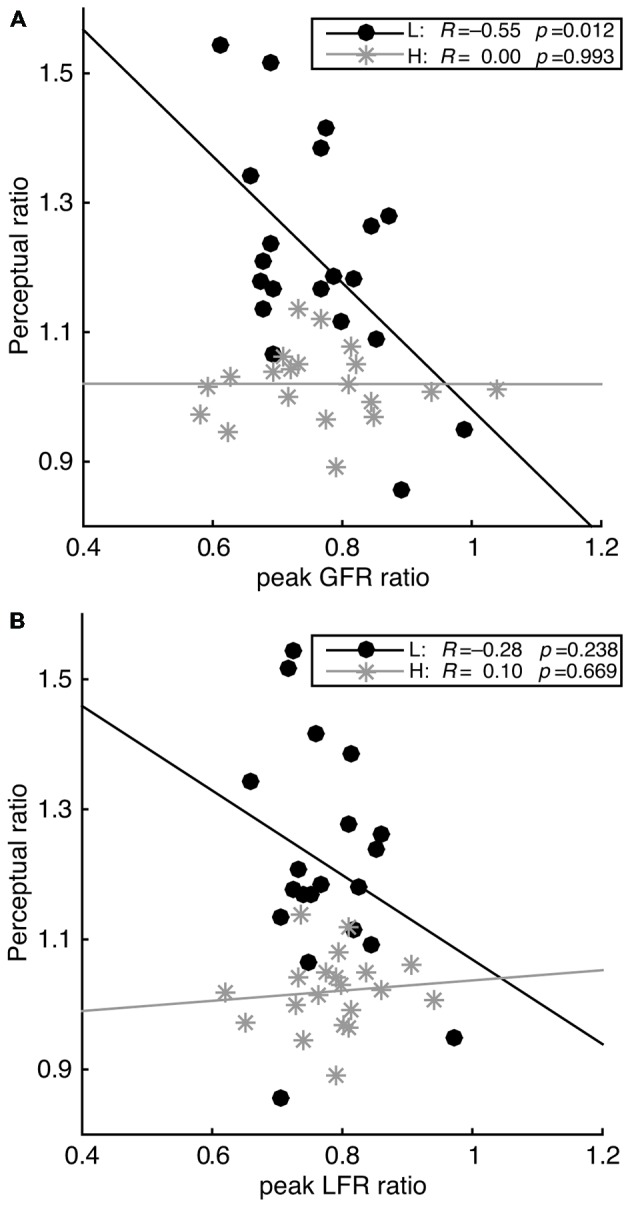
**Regression lines between the perceptual estimate ratio and the peak grip force ratio (A: peak GFR) and the peak load force ratio (B: peak LFR).** Correlations are shown separately for light (L, black circles) and heavy objects (H, light gray asterisks). Correlation coefficients and *p*-values are indicated in the captions. Note that for light objects, but not heavy, the weight perception bias was larger as the magnitude of the sensorimotor memory effect increased.

The between-subject correlation of the sensorimotor memory effect and the perceptual bias is shown in Figure 4. This correlation was calculated for the pooled measurements of Experiments 1a and 1b. For light lifts, significant correlations were found between the perceptual ratios and the peak GFR ratios (*R* = −0.55, *p* = 0.012), but not for the peak LFR ratios. For heavy lifts, no significant correlation was found.

To test whether a larger sensorimotor memory effect was indeed produced with longer sequences of the same weight, two-trial sequences were compared with three-trial sequences. To do this, ratios of lifts preceded by heavy and light objects were compared within Experiment 1b, for *light* (L*L*/H*L* vs. LL*L*/HH*L*) and *heavy* (L*H*/H*H* vs. LL*H*/HH*H*) lifts separately. For light lifts, sensorimotor memory effects in the three-trial sequences were larger than in the two-trial sequences (peak GFR: *t*_(9)_ = −3.46, *p* = 0.007, peak LFR: *t*_(9)_ = −4.64, *p* = 0.001, GF at peak GFR: *t*_(9)_ = −3.32, *p* = 0.009), although this did not reach significance for LPD (*t*_(9)_ = 2.13, *p* = 0.062). Interestingly, the perceptual weight bias was also larger for the three-trial sequence compared to the two-trial sequence (*t*_(9)_ = 2.57, *p* = 0.030). For heavy lifts, the sensorimotor memory effects were also larger in the three-trial compared to the two-trial sequences (peak GFR: *t*_(9)_ = −3.75, *p* = 0.005, peak LFR: *t*_(9)_ = −4.97, *p* = 0.001, LPD: *t*_(9)_ = 8.80, *p* < 0.001, GF at peak GFR: *t*_(9)_ = −2.68, *p* = 0.025). However, no significant difference was found for perceptual estimates (*t*_(9)_ = 0.67, *p* = 0.520). Altogether, this experiment shows that larger sensorimotor memory effects on force scaling lead in turn to larger weight perception biases, which suggests a tight link between the action planning and perception.

### Experiment 2: Weight History Does Not Affect Passive Weight Perception

**Figure 5:**
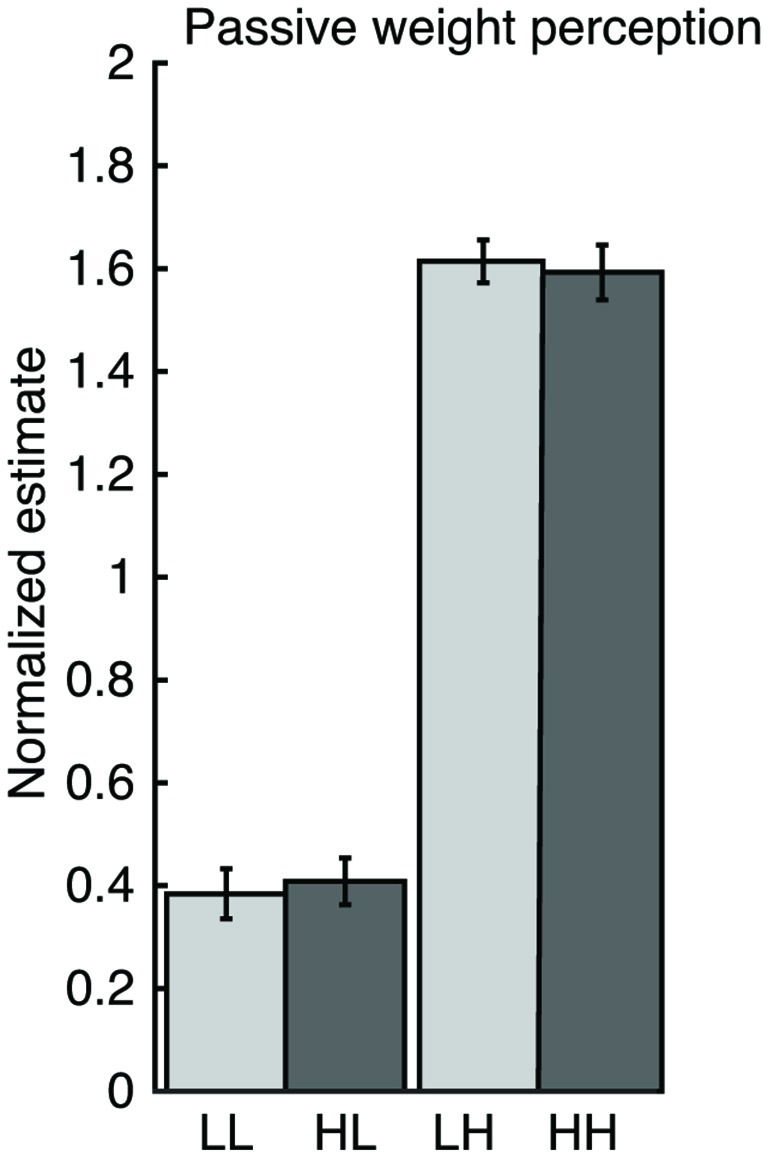
**Results of Experiment 2.** Weight sequence did not affect normalized perceptual estimates when object weight was passively presented onto the subjects’ right hand.

When participants were presented with different forces (1 or 5 N) on their resting hand, no significant differences in perceptual estimates of the current object weight were seen when trials were preceded by heavy compared to light weights (light: *t*_(7.0)_ = −0.69, *p* = 0.513, heavy: *t*_(7.0)_ = 0.31, *p* = 0.769; Figure 5). The lack of effect in this control experiment highlights the lifting motion as the key component for biasing weight perception.

## Discussion

The aim of this study was to evaluate the interaction between object lifting and weight perception. Specifically, we investigated the relationship between sensorimotor memory effects and weight estimation in an object grip-lift task. We asked participants to lift light or heavy objects and estimate their weight. Importantly, the order in which light and heavy weights were presented was unpredictable. In short, we found that not only fingertip forces but also perceptual estimates were influenced by the previous lift. This finding indicates that action parameters and perception are intimately linked. Since sensorimotor memory has been considered as a fast 1-trial learning process (e.g., Fu et al., 2010), this shows how learning novel object dynamics can affect perceptual object representations. In accordance with previous studies (Johansson and Westling, 1988; Loh et al., 2010), we found a sensorimotor memory effect when participants had to lift a series of different objects. Fingertip forces were planned according to the previous lift and this effect was present for both light and heavy objects. This result indicates that no default strategy was used for lifting objects, but that forces were scaled based on recent experience. Importantly, besides the effect of the lifting order on force scaling, a bias was also found for perceptual weight estimations in both Experiments 1a and 1b. When a light object was lifted after a heavy object, it was perceived to be lighter than when lifted after a light object. To test whether the perceptual bias did not merely result from a cognitive contrast effect independent of active lifting or force scaling, a control experiment was performed in which a force was passively exerted with a haptic device on the hand at rest. In this case, we did not find any perceptual bias depending on the previous felt force.

The observation that the perceptual bias is only present when actively lifting an object suggests that the estimation bias is related to force scaling. Indeed, this relationship was found both within individual subject data and across all participants. This correlation was negative: lower weight estimations were associated with higher force rates. In addition, increasing the magnitude of sensorimotor memory effects by lengthening the trial history (Experiment 1b) also enhanced the perceptual bias. Although the within-subject correlations showed a significant relationship between perceptual estimates and both grip and load forces, the between-subject correlations were only significant for grip but not load force. This stronger relationship for grip force could be explained by a dissociable neural control of grip and load forces (Davare et al., 2006, 2007), likely to have different impacts on brain areas involved in perception.

The bias for perception was only seen for light lifts but not heavy ones, while a sensorimotor memory effect was present for lifts of both object weights. Although the peak force rates were found to be a significant covariate for the perceptual estimates with the lifting of a heavy object in Experiment 1b, there were still few within-subject relationships and no between-subject correlations between sensorimotor memory and perceptual biases. There are two possible explanations for the absence of perceptual bias for heavy objects. First, the force rate differences might not be large enough to produce perceptual differences in heavy weights. Perceptual differences of weight behave according to Weber’s law. This means that the just noticeable difference is related to the intensity of the stimulus. Consequently, larger weight differences are needed with higher values to be able to be perceived. The magnitude of the sensorimotor memory effect was similar for both heavy and light objects, as seen in the difference in grip force at peak GFR (around 0.5–0.8 N). However, this difference is relatively much larger compared to a light (2 N) than to a heavy (6 N) object. Therefore, the magnitude of the sensorimotor memory effect might not have been salient enough to bias perception of a heavy weight, which was therefore perceived as having the same weight independent of the lifting history. A second explanation relies on the loading phase being much longer for the heavy object. When lifting a heavy weight after a light one, the planned forces are too small and lift-off does not occur when expected. Consequently, forces keep increasing at the same rate as for a light lift until lift-off takes place, a process during which feedback loops are heavily involved (Johansson and Flanagan, 2009). These recurrent feedback loops over the course of a longer loading phase might also influence weight perception and minimize the estimation bias. When a light object is lifted after a heavy one, feedback mechanisms are also used to correct the force overshoot and stabilize the object. However, in this case the stabilization process occurs after lift-off and might be less influential on the weight perception.

**Figure 6:**
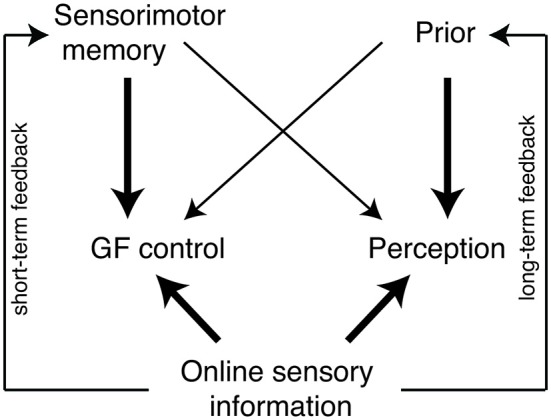
**Schematic diagram of the influence of sensorimotor memory, priors and online sensory information on grip force (GF) control and perceptual estimation.** The arrow thickness reflects the importance of the gain of one input. Two feedback loops represent the input of sensory information used to build up the sensorimotor memory and the prior.

All in all, these results show that both perceptual and force parameters are affected by previous object lifts and that these parameters are also correlated. The finding of the association between perceptual estimates and force scaling appears to contrast studies on the size-weight illusion, where these two control systems seem to be dissociated. In previous research, it was found that perception of object weight was influenced by object size, whereas force scaling was not (Flanagan and Beltzner, 2000; Grandy and Westwood, 2006). In those studies, sensorimotor memory did not affect weight perception. Figure 6 provides a schematic explanation for both of these findings. Online sensory information from the current object provides inputs to control forces applied by the fingertips and perceptual weight estimation. Furthermore, online information is also used in feedback loops to build up sensorimotor memory and priors. These loops reflect short and long-term learning processes of object representations. The sensorimotor memory is the representation that is build up from previous experience with the current or a similar object. This can be formed after a single trial and is therefore a short-term feedback loop. A prior is a long-term learned association between two object properties, which requires more time to develop, but also lasts longer. For example, a size-weight prior states that large objects are heavy assuming a constant density. In the diagram, sensorimotor memory and priors both influence the control of grip force and weight perception, but to a different extent. Sensorimotor memory has a stronger influence on force control than on perceptual weight estimation. Conversely, the prior has a stronger influence on perception than on force control. In the current experiment, no sizes cues or other priors are available, so only the sensorimotor memory influences the grip force control as well as the perceptual estimates. In the case of the size-weight illusion, a prior influences the grip force and perception. For the first trials in a size-weight illusion setting, no sensorimotor memory is build up yet and grip force scaling is affected by the prior (Gordon et al., 1991; Flanagan and Beltzner, 2000). After a few lifts, the sensorimotor memory of the object weights dominates the grip force control. For the perceptual estimation, the prior dictates weight estimation and produces the persisting size-weight illusion.

The effects of sensorimotor memory or priors on force control and perception do not only have a different gain, but also influence force and perception in opposite directions. Whereas force rates are scaled according to a weight prediction, weight perception changes based on the motor correction required when there is a mismatch between expected and actual weight. In other words, the force and perceptual parameters are affected by trial history in an opposite way: although *higher* force rates are used to lift an object after a heavy lift, it is actually perceived to be *lighter*. This negative relationship between forces and estimates suggests that a sense of effort is perceived. When lifting a light object after a heavy lift, less effort is needed than originally planned. The correction of the predicted weight compared to the actual weight makes the object to be perceived lighter than expected. Note that the weight expectation is an implicit phenomenon in the present experiments, and only results from sensorimotor memory-driven changes in force scaling. This is in contrast to explicit expectations that can also lead to different weight perceptions (e.g., in the size-weight illusion, Buckingham and Goodale, 2010).

Previous research relating grip forces and weight perception also point to the perception of a sense of effort. These findings reflect the sense of effort needed in the static phase of lifting, where a higher needed grip force (more effort) is associated with a higher perceptual estimate (Flanagan et al., 1995; Flanagan and Bandomir, 2000). In contrast to these studies, the objects lifted or the way they were lifted did not differ between conditions in the present experiments, but only the history and the planning of the action. If weight ratings would be estimated based on the static holding phase, where grip and load forces were the same in all cases, perception should then be the same. However, the dynamic phase of the lift differed according to lifting history. Hence, weight estimation seems to be formed early in the lift or is at least influenced by this phase. This is the first study showing an effect of the dynamic lifting phase, i.e., GFRs, on perception. Since the GFRs in the dynamic phase of the lift reflect the planning of the lift, this indicates that the action plan has an impact on the perception of an object.

The effect of force control on weight perception can generate several other predictions based on other findings related to sensorimotor memory. For instance, it has been found that sensorimotor memory is only partly disrupted by an isometric contraction (Cole et al., 2008), affecting grip force but leaving load force unchanged. It is therefore plausible to assume that perception of object weight should be altered by an isometric contraction, similar to a conscious grip force increase (Ellis and Lederman, 1999). Another interesting study found that with a series of increasing weights, force prediction does not depend on the last lift, but is extrapolated from the series (Mawase and Karniel, 2010). Given this extrapolation-driven increase in force scaling, we expect even larger changes in perception of object weight. Finally, as sensorimotor memory can be transferred between hands (Gordon et al., 1994; Nowak et al., 2005b), perceptual biases might also be found when alternating lifts with the two hands.

Future research should aim to find the neural substrate underlying the effect of sensorimotor memory on weight perception. It is plausible that this effect does not stem from a single brain area, but involves a network of areas; the primary motor cortex (M1), cerebellum and lateral occipital complex (LOC) are likely to be the key nodes in this network (van Polanen and Davare, 2015). The role of M1 in building up sensorimotor memory has previously been demonstrated (Chouinard et al., 2005; Nowak et al., 2005b; Loh et al., 2010). However, it has recently been shown that M1 also plays a role in sense of effort (Takarada et al., 2014). It is believed that a sense of effort is formed through both peripheral (Luu et al., 2011) and central (Morree et al., 2012) inputs. In our study, the discrepancy between the anticipated sensory consequences and perceived signals seems to have an impact on perceptual responses. In fact, this effect is proportional to the amount of force correction required. The cerebellum is proposed to play a role in predictive motor control and in the comparison between predicted and actual motor states (Nowak et al., 2007). The sensory consequences are predicted based on internal models (Wolpert and Flanagan, 2001) which are believed to reside in the cerebellum (Kawato et al., 2003). This structure is also involved in the control of fingertip forces and sensorimotor memory (Nowak et al., 2005a). In addition, Jenmalm et al. (2006) have found that processing of weight switches was different for light lifts preceded by heavy objects than for heavy lifts preceded by light objects. Increased BOLD signal was found in M1 for conditions with an increase in weight (light to heavy switch) and in the cerebellum for conditions with a decrease in weight (heavy to light switch). Interestingly in our study, we have only found perceptual biases for heavy to light switches, suggesting a possible role of the cerebellum in mediating this effect. Finally, it has recently been discovered that object weight representations are also found in the LOC (Gallivan et al., 2014). The role of LOC in the multimodal recognition of objects (Amedi et al., 2001) makes this area a possible site for perceptual weight estimation.

To summarize, we used sensorimotor memory as a tool to manipulate implicitly subjects’ expectations about the weight of an object. Importantly, we found that the previous lift biased weight perception and this effect was negatively correlated with the magnitude of the planned force parameters. This highlights a key role of the action plan in modulating perception: if there is a mismatch between predicted and actual object weight, the implementation of online force corrections will also influence weight perception in a proportional manner.

## Author Contributions

VVP and MD designed the experiment. VVP performed the data acquisition. VVP and MD analyzed and interpreted the data. VVP and MD drafted and revised the manuscript and approved the final version. VVP and MD agree to be accountable for all aspects of the work in ensuring that questions related to the accuracy or integrity of any part of the work are appropriately investigated and resolved.

## Conflict of Interest Statement

The authors declare that the research was conducted in the absence of any commercial or financial relationships that could be construed as a potential conflict of interest.
